# Current Age, Age at First Sex, Age at First Homelessness, and HIV Risk Perceptions Predict Sexual Risk Behaviors among Sexually Active Homeless Adults

**DOI:** 10.3390/ijerph15020218

**Published:** 2018-01-26

**Authors:** Diane Santa Maria, Daphne C. Hernandez, Katherine R. Arlinghaus, Kathryn R. Gallardo, Sarah B. Maness, Darla E. Kendzor, Lorraine R. Reitzel, Michael S. Businelle

**Affiliations:** 1 Department of Nursing Systems, School of Nursing, University of Texas Health Science Center, Houston, TX 77030, USA; 2 Department of Health and Human Performance, University of Houston, Houston, TX 77204, USA; dherna32@Central.UH.EDU (D.C.H.); krarling@Central.UH.EDU (K.R.A.); 3 Department of Psychological, Health, and Learning Sciences, University of Houston, Houston, TX 77204, USA; lrreitzel@uh.edu; 4 Department of Health Promotion and Behavioral Sciences School of Public Health, University of Texas Health Science Center, Houston, TX 77030, USA; Kathryn.R.Gallardo@uth.tmc.edu; 5 Department of Health and Exercise Science, University of Oklahoma, Norman, OK 73019, USA; smaness@ou.edu; 6 Health Sciences Center, University of Oklahoma, Oklahoma City, OK 73104, USA; Darla-Kendzor@ouhsc.edu (D.E.K.); Michael-Businelle@ouhsc.edu (M.S.B.)

**Keywords:** sexual behaviors, homeless adults, HIV, onset of homelessness, age

## Abstract

While HIV disproportionately impacts homeless individuals, little is known about the prevalence of HIV risk behaviors in the southwest and how age factors and HIV risk perceptions influence sexual risk behaviors. We conducted a secondary data analysis (*n* = 460) on sexually active homeless adults from a cross-sectional study of participants (*n* = 610) recruited from homeless service locations, such as shelters and drop-in centers, in an understudied region of the southwest. Covariate-adjusted logistic regressions were used to assess the impact of age at homelessness onset, current age, age at first sex, and HIV risk perceptions on having condomless sex, new sexual partner(s), and multiple sexual partners (≥4 sexual partners) in the past 12 months. Individuals who first experienced homelessness by age 24 were significantly more likely to report condomless sex and multiple sexual partners in the past year than those who had a later onset of their first episode of homelessness. Individuals who were currently 24 years or younger were more likely to have had condomless sex, new sexual partners, and multiple sexual partners in the past 12 months than those who were 25 years or older. Those who had low perceived HIV risk had lower odds of all three sexual risk behaviors. Social service and healthcare providers should consider a younger age at homelessness onset when targeting HIV prevention services to youth experiencing homelessness.

## 1. Introduction

According to the National Alliance to End Homelessness (NAEH), over 500,000 people are homeless on any given night in the U.S. [1]. Other reporting strategies indicate that as many as 1.7–2.5 million youth under age 25 years are homeless or unstably housed on any given night in the U.S. [2,3,4]. Individuals who experience homelessness are at greater risk of acquiring or transmitting HIV compared with people in stable housing [5], with HIV prevalence rates among the homeless being nine times higher than in the general population [6]. Despite this, those who experience homelessness have dramatically limited access to HIV prevention programs, particularly pre-exposure prophylaxis (PrEP) and non-occupational post-exposure prophylaxis (nPEP), two promising biomedical HIV prevention strategies. Homeless youth are less likely to enroll in HIV PrEP trials with one study reporting that only 1% of HIV-negative participants were prescribed PrEP [7]. In PrEP trials, fewer young adults have enrolled than older adults [8] and uptake [7] and adherence were lower in those with unstable housing [9].

Homelessness is associated with several HIV risk behaviors, such as having condomless sex and multiple sexual partners, which contribute to disparities in sexual health outcomes. For example, condomless sex is a prevalent HIV risk behavior among individuals experiencing homelessness. In studies of homeless men, over two-thirds of participants reported having condomless sex in the past six months [10] and each additional sex partner has been found to be associated with an increase of more than two times the odds of engaging in condomless sex [11]. In a sample of both male and female sexually active homeless adults, approximately 76% reported having condomless sex [7]. Among younger homeless populations, 60% reported condomless sex during their last sexual encounter [12]. Among heterosexual homeless men, the strongest predictors of condom use were attitudes about condom use, self-efficacy for condom use, partner type (i.e., long-term or casual), and partner communication about condom use [13]. In addition, having multiple sexual partners and depression were found to be associated with consistent condom use [10]. Predictors of condom use among homeless women include condom efficacy (i.e., belief that condoms reduce risk) and their perceived risk of getting HIV. For example, women who believe they have low HIV susceptibility are less likely to have condomless sex [14]. Yet, in other homeless adult samples, HIV susceptibility, operationalized as worry about getting infected with HIV or AIDS, was not found to be significantly associated with condom use [11]. While researchers have identified several common predictors of condom use among various homeless subgroups, the relation between other factors such as HIV risk perceptions, current age, age at onset of sex and first period of homelessness remains unclear.

Other HIV risk behaviors such as having multiple sexual partners and concurrent sexual partners are prevalent among homeless populations, including ex-offenders and street-involved youth [15,16]. In a study of homeless men from Los Angeles [17], almost 40% reported multiple concurrent sex partners, i.e., having sex with more than one person around the same time, which is a known risk factor for HIV [18]. Finally, sexually active homeless individuals reported more sex partners in their lifetime and in the past 12 months with an unknown HIV serostatus partner compared with HIV+ housed adults [19].

Subgroups of homeless individuals report high rates of sexual risk behaviors including females, young adults under 25 years old, sexual minorities, and those with early sexual debut [20,21]. Homeless women are more likely than homeless men to engage in sexual risk behaviors, such as having condomless sex with casual partners and having multiple partners [22,23,24,25,26]. Homeless youth are 6–12 times more likely to become infected with HIV than housed youth, with prevalence rates as high as 13% [27,28]. Homeless youth have earlier sexual debut; are more likely to have multiple partners, trade sex for food, shelter, money, or substances, and use substances before sex; and are less likely to use a condom or contraception than stably housed youth [20,29,30,31,32,33]. Lesbian, gay, bisexual, transgendered, and questioning (LGBTQ) homeless persons have a higher risk of HIV than heterosexual individuals. Among a sample of homeless LGBTQ young adults, 17% reported a diagnosis of HIV [33]. As well, LGBTQ homeless youth experience higher rates of sexually transmitted infections (STIs) than homeless heterosexual youth [34]. Finally, both males and females who had sex before age 13 years were more likely than non-early sexual initiators to have multiple sexual partners during their lifetime and to engage in condomless sex [35].

Multiple risk factors for engaging in sexual risk behaviors have been identified among homeless populations. However, less is known about differences in sexual risk behaviors according to current age (i.e., young adults vs. older adults) as many studies of homeless youth do not compare youth to older adults in the same sample. Less is known as well about the age of homelessness onset as a potential, independent risk factor despite findings suggesting that age of onset is correlated with other risk behaviors such as substance use, with a younger age at the onset of homelessness being associated with a higher incidence of substance use [36]. An Oakland, California based study found that, among homeless adults >50 years old, those who first became homeless before age 50 reported higher rates of substance use than those with a later age of homelessness onset [37]. While research demonstrates that age at the onset of homelessness is associated with substance use, little is known about the relation between age at the onset of homelessness and sexual risk behaviors in a broader sample of homeless adults and young adults.

## 2. Purpose

In this study, we examined the prevalence of sexual risk behaviors—having condomless sex, a new sexual partner, and multiple sexual partners—among sexually active homeless adults in Oklahoma City, OK, USA. In addition, we assessed the relations between HIV risk perception, age at sexual debut, age at the onset of homelessness, current age, and sexual risk behaviors. We hypothesized that those who were younger than 25 years and those who experienced homelessness at an earlier age (i.e., under 25 years old) would report more sexual risk behaviors and that those with low perceived HIV risk would report fewer sexual risk behaviors.

## 3. Methods

### 3.1. Data and Sample

From July–August 2016, participants were recruited through flyers posted at six different homeless shelters in Oklahoma City, OK, USA. To be enrolled, participants must have been 18 years of age or older, receiving services (e.g., shelter, counseling) at the targeted shelters, and had a minimum 7th grade English literacy level based on a score of 4 or higher on the Rapid Estimate of Adult Literacy in Medicine-Short Form (REALM-SF) [38]. After the screening and informed consent process, each participant was given a questionnaire to complete on a tablet computer, which enabled the participant to see survey items on the screen and hear the questions being read aloud via headphones. Participants took about 1 h to complete the survey and were compensated for their time with a $20 department store gift card. The Institutional Review Boards at the (the University of Texas Health Science Center, the University of Houston, and the University of Oklahoma Health Sciences Center approved this study.

A total of 648 participants were screened for inclusion in the parent study. Thirty-four participants were excluded because they did not meet the reading level criteria REALM literacy score. Four eligible people chose not to participate. A total of 610 adults were enrolled and completed study measures over the 12 day data collection period across 6 shelter sites. An additional 21 participants were not literally homeless and therefore were excluded from the analysis. Of the sexually active participants (*n* = 467), none had missing data on any of the sexual risk behavior measures (i.e., dependent variables). However, 3 participants were excluded for having missing data on independent variables and 4 additional participants were excluded for missing data on the covariates. The final analytic sample for this study included 460 homeless and sexually active participants.

The subsample of sexually active participants in this study differed from those excluded who were not currently sexually active. The sexually active participants were younger, minority race, and had less than a high school degree. The sample for this study also demonstrated more moderate to severe stress and were more often diagnosed with alcohol or substance use disorder compared to the not sexually active participants from the parent study.

### 3.2. Measures

#### 3.2.1. Dependent Variables

Condomless sex. Participants were asked how often they had vaginal or anal sex without using a condom in the past 12 months. Participants who responded that they had sex without using a condom less than half of the time, about half of the time, not always but more than half the time, or always were coded as having condomless sex, while participants who responded that they never engage in sex without a condom were coded as always having sex with a condom.

New sexual partner. To determine if a participant had a new sexual partner, participants were asked, “Did you have any kind of sex with a person that you have never had sex with before in the past 12 months?” (yes/no).

Multiple sexual partners. Participants were asked to report the number of people they had any kind of sex with in the past 12 months. Responses were dichotomized to 4 or more partners or fewer than 4 to align with the literature on multiple sexual partners and allow for comparisons with other studies and populations [7,16].

Sexually Transmitted Infection (STI). Participants were asked if a health care professional had ever told them they had genital herpes, genital warts, Human papillomavirus or HPV, gonorrhea (sometimes called GC or clap), Chlamydia, or Syphilis.

#### 3.2.2. Independent Variables

The independent variables included in the model represented sexual activity and HIV risk perceptions, homelessness and age characteristics, stress, and substance use. 

HIV risk perceptions. Participants were asked to rate their perception of their HIV risk from 0 (No risk) to 5 (High risk) [39]. Participants who responded as somewhat at risk, moderate risk, or high risk were coded as high perceived risk, while participants who responded as low or no risk were coded as low perceived risk. 

Age characteristics. Participants reported their age at their first sexual encounter. This variable was dichotomized to either <14 years or ≥14 years of age to align with sexual initiation surveys [40]. Participants reported their age at the onset of homelessness, which was dichotomized as <25 years or ≥25 years to align with the literature on homeless youth that often includes youth 25 years old and younger [41,42]. Participants also reported their current age, which was dichotomized as <25 years or ≥25 years.

#### 3.2.3. Covariate Measures

The regression models included various characteristics that may influence participants’ sexual risk behaviors, in addition to HIV risk perceptions and age characteristics. Covariates included sex (1 = female; 0 = male), marital status (1 = married; 0 = not married), education (1 = high school diploma or more; 0 = less than high school diploma), race/ethnicity (1 = minority race/ethnicity; 0 = white), history of sexually transmitted infections (1 = present; 0 = absent), and sexual orientation (1 = LGBTQ; 0 = heterosexual). The four-item Perceived Stress Scale was used to assess perceived stress [43]. Items were summed, and responses were grouped into two groups: moderate to severe stress (score of 9 or more) and low stress (score less than 9) [44,45]. Participants were also asked if they had ever received a diagnosis for alcohol or substance use disorder (yes/no).

### 3.3. Analytic Plan

Descriptive and logistic regression analyses were conducted using STATA version 24.0 statistical software (StataCorp. LP, College Station, TX, USA). Bivariate analyses assessing the relation between sexual risk behaviors and individual factors were conducted using chi-square tests. Three covariate-adjusted logistic regression models were conducted, one for each sexual risk behavior (i.e., condomless sex, new sexual partners, and more than 4 sexual partners in the past 12 months). Standard errors were adjusted to account for the lack of independence of observations, as participants were clustered within the shelter location where they completed the survey [46].

## 4. Results

### 4.1. Sample Characteristics

Participants (*n* = 460) were predominantly ≥25 years old (94%), male (61%), single (88%), white (60%), and heterosexual (92%), and most had at least a high school diploma (76%) (Table 1). This sample approximates the age and sex of the homeless population surveyed in the Oklahoma City Point-in-Time count in 2016 [47].

Regarding the prevalence of sexual risk behaviors in the past 12 months, 53% of the participants reported engaging in condomless sex, 35% had a new sexual partner, and 12% had multiple sexual partners. Eighty-nine percent of participants perceived themselves to be at low risk for HIV. Seventy-one percent of the participants initiated sexual activity at 14 years or older ($\bar{X}$ = 14.80 ± 3.67 years). Most participants (70%) were ≥25 years old at the onset of homelessness ($\bar{X}$ = 32.6 ± 13.19 years) and had been homeless for an average of 1.71 ± 2.64 years (median = 9.06 months). Additionally, 22% report a positive sexually transmitted infection history.

The results of the bivariate analyses are shown in Table 1. Having low perceived HIV risk was independently associated with lower prevalence of all three sexual risk behaviors. An early sexual debut (i.e., initiating sexual activity at 13 years or younger) was associated with having condomless sex but not with having new or multiple sexual partners in the past 12 months. Experiencing first homelessness at 24 years or younger and current age of 24 years old or younger were associated with greater odds of having condomless sex, a new sexual partner, and multiple sexual partners in the past 12 months. Early sexual debut was also associated with having low perceived HIV risk, being female, being married, having less than a high school diploma, and being a racial/ethnic minority. Homelessness onset at <25 years old was associated with having low perceived HIV risk, currently being 24 years or younger, having completed less than a high school diploma, and being a racial/ethnic minority. Currently being younger than 25 years old was associated with being LGBTQ. No significant associations were found between the age characteristics and perceived stress or alcohol and substance use disorder diagnoses.

### 4.2. Regression Analyses

Predictors of Condomless Sex. In the covariate-adjusted models, low perception of HIV risk was associated with 73% lower odds of engaging in condomless sex in the past 12 months (Table 2). Being female, married, and having a sexually transmitted infection were respectively associated with a 110%, 283%, and 107% higher odds of having condomless sex.

Predictors of Having a New Sexual Partner. Low perception of HIV risk was associated with 71% lower odds of having a new sexual partner in the past 12 months. Being younger than 25 years was significantly associated with 265% higher odds of having a new sexual partner. Being married was also associated with 55% lower odds of having a new sexual partner while having a diagnosis of alcohol or substance use disorder was found to be associated with a 49% higher odds of having a new sexual partner in the past 12 months.

Predictors of Having Multiple Sexual Partners. Low perception of HIV risk was associated with 77% lower odds of having multiple sexual partners in the past 12 months. Being younger than 25 years was significantly associated with 108% higher odds of having multiple sexual partners in the past 12 months. Experiencing homelessness before 25 years old was associated with 80% higher odds of having multiple sexual partners in past 12 months. Additionally, identifying as LGBTQ was associated with 123% higher odds of having multiple sexual partners in the past 12 months, while a diagnosis of alcohol or substance use disorder was found to be associated with a 32% lower odds of having multiple sexual partners in the past 12 months.

## 5. Discussion

Among a cross-sectional sample of sexually active homeless adults from an understudied region in the southwestern United States, we examined the prevalence of recent sexual risk behaviors, including having condomless sex, new sexual partners, and having multiple sexual partners in the past year. We found lower rates of condomless sex than have been found in other studies of homeless adults [7,10]. We also found that the proportion of individuals who had condomless sex, new sexual partners, and multiple sexual partners in the past 12 months was higher among those younger than 25 years old than among those 25 years and older. This aligns with the literature that homeless youth have significant sexual health risks and high prevalence of sexual risk behaviors [19,29,30,31,32]. Findings from this study add to the literature on sexual risk behaviors among homeless populations by comparing homeless youth and older adults within the same sample and suggesting that the age at the onset of homelessness is an important, independent risk factor to consider when addressing sexual risk behaviors, even after controlling for current age.

In contrast to other studies that have found no significant association with perceived HIV risk and sexual risk behaviors [11], in this sample, we found that having low HIV risk perception was associated with significantly reduced risk of having condomless sex, a new sexual partner, and multiple partners in the past year. This may be related to the participant’s ability to accurately conduct a self-assessment of their HIV risk behaviors, i.e., those with low risk behaviors conclude they are at low risk for HIV and therefore report low HIV risk perceptions.

Sex, marital status, ever having an STI, sexual orientation, and alcohol/substance use disorders were found to be significantly related to sexual risk behaviors. Females reported more condomless sex, which aligns with the literature among homeless populations [22,33]. However, in contrast to the literature, fewer females reported having multiple sexual partners [22,23,24,26]. As expected, being married was found to be significantly associated with reduced odds of new sexual partners and increased odds of having condomless sex. This aligns with the literature that has revealed lower condom use among steady sexual partners and may be related to low HIV risk perception among those having sex within established relationships [48]. Those reporting ever having an STI had increased odds of condomless sex and those who identify as LGBTQ had increased odds of having multiple sexual partners. We also found that while having a diagnosed alcohol or substance use disorder increased the odds of reporting a new sexual partner in the past 12 months, it decreased the odds of having multiple sexual partners. This suggests that it may be important to also screen for substance use and assist with access to substance abuse treatment in conjunction with HIV risk prevention counseling, particularly among homeless persons with early homelessness onset.

Study strengths include the novel analysis of age at the onset of homelessness and current age as potential factors contributing to sexual risk behaviors among a large sample of both younger and older homeless adults. There are several limitations of this study. The sample was collected in one understudied southwestern region of the United States; therefore, the results may not be generalizable to other homeless adult populations. This sample was predominantly ≥25 years old, white, and heterosexual which does not approximate the demographics of many other homeless populations. This study is also limited by reliance upon self-report data and the cross-sectional design. Thus, we can interpret associations but not causation from these data. The sample represents only homeless adults interfacing with shelter services. Because this study was not designed to include street outreach to homeless adults disconnected from service providers, the sexual health risk factors may be an underrepresentation of all homeless adults, both connected and disconnected from service providers such as shelters and drop-in centers. The lower prevalence of sexual risk behaviors may also reflect the older age of the sample, as homeless youth report higher prevalence of sexual risk behaviors [9]. As well, multiple analyses were conducted which increases the risk type 1 errors. Finally, participants recruited for this study were from homeless adult vs. homeless youth serving organizations.

## 6. Conclusions

Current age and age at the onset of homelessness should be considered in planning sexual health programs and HIV/STI prevention interventions among homeless populations. Social service and healthcare providers should consider screening homeless adults for the age at the onset of homelessness as a gauge for sexual risk behaviors. Youth experiencing homelessness and those who experience early homelessness engage in more HIV risk behaviors than older homeless individuals and those who first experience homelessness later in their adult life. To effectively scale-up HIV prevention among homeless youth, health and social systems should increase access to free HIV prevention counseling that includes linkages to care and patient navigation, refs promoting condom use and HIV prevention strategies including PrEP and nPEP, and providing free condoms, HIV/STI screening, and treatment in locations that are easily accessible by public transportation.

## Figures and Tables

**Table 1:** Sample Characteristics by Sexual Risk Behaviors, % (*n*) or Mean (SD).

Variable	Analytic Sample (*n* = 460)	HIV Risk Perceptions	Age Characteristics		
		Low Perceived HIV Risk (*n* = 411)	First Sex by ≤13 Years Old (*n* = 132)	Age at First Homeless Was ≤24 Years (*n* = 139)	Current Age ≤24 Years (*n* = 28)
Sexual Risk Behaviors					
Condomless Sex					
Sex with a Condom	47% (218)	51% (208)	40% (53)	37% (52)	25% (7)
Sex without a Condom	53% (242)	49% (203) ***	60% (79) *	63% (87) **	75% (21) *
New Sexual Partners					
No	65% (301)	69% (283)	65% (86)	58% (80)	36% (10)
Yes	35% (159)	31% (128) ***	35% (46)	42% (59) *	64% (18) **
Multiple Sexual Partners					
≤3 Sexual Partners	88% (403)	90% (371)	86% (113)	80% (111)	71% (20)
≥4 Sexual Partners	12% (57)	10% (40) ***	14% (19)	20% (28) **	29% (8) **
HIV Risk Perceptions					
Low Perceived HIV Risk	89% (411)	100% (411)	84% (111) *	83% (116) **	82% (23)
High Perceived HIV Risk	11% (49)	--	16% (21)	17% (23)	18% (5)
Age Characteristics					
Age of First Sex	14.80 (3.67)	14.93 (3.57)	10.71 (2.81)	14.42 (3.29)	14.86 (2.51)
≤13 years	29% (132)	27% (111) *	100% (132)	32% (44)	29% (8)
≥14 years	71% (328)	73% (300)	--	68% (95)	71% (20)
Age at First Homeless	32.67 (13.19)	33.12 (13.14)	30.49 (12.88)	17.73 (4.63)	19.21 (3.24)
≤24 years	30% (139)	28% (116) **	33% (44)	100% (139)	100% (28) ***
≥25 years	70% (321)	72% (295)	67% (88)	--	--
Current Age	43.03 (12.24)	43.52 (12.21)	43.36 (11.27)	36.40 (12.52)	21.54 (1.88)
≤24 years	6% (28)	6% (23)	6% (8)	20% (28) ***	100% (38)
≥25 years	94% (432)	94% (388)	94% (124)	80% (111)	--
Covariates					
Sex					
Male	61% (281)	61% (252)	70% (93)	64% (89)	71% (20)
Female	39% (179)	39% (159)	30% (39) **	36% (50)	29% (8)
Marital Status					
Not Married	88% (406)	88% (362)	83% (109)	90% (125)	86% (24)
Married	12% (54)	12% (49)	17% (23) *	10% (14)	14% (4)
Education					
Less than High School Diploma	24% (109)	23% (95)	32% (42) **	35% (49) ***	36% (10)
High School Diploma or more	76% (351)	77% (316)	68% (90)	65% (90)	64% (18)
Race/ethnicity					
White	60% (274)	61% (252)	52% (69)	53% (73)	61% (17)
Minority Race/Ethnicity	40% (186)	39% (159) *	48% (63) *	47% (66) *	39% (11)
Sexually Transmitted Infections					
Present	22% (101)	21% (86)	26% (34)	21% (29)	7% (2)
Absent	78% (359)	79% (325)	74% (98)	79% (110)	93% (26)
Sexual Orientation					
Heterosexual	92% (422)	93% (381)	92% (121)	88% (123)	79% (22)
LGBTQ	8% (38)	7% (30) *	8% (11)	12% (16)	21% (6) **
Perceived Stress	7.75 (3.60)	7.60 (3.61)	7.99 (3.86)	7.87 (3.71)	7.86 (3.79)
Low Stress	60% (278)	62% (256)	58% (77)	58% (81)	50% (14)
Moderate to Severe	40% (182)	38% (155) *	42% (55)	42% (58)	50% (14)
Alcohol or Substance Use Disorder Diagnoses					
No	61% (279)	61% (252)	58% (77)	66% (92)	64% (18)
Yes	39% (181)	39% (159)	42% (55)	34% (47)	36% (10)

HIV = Human Immunodeficiency Virus; LGBTQ = lesbian, gay, bisexual, transgender, questioning. Significance of chi-square tests between each sexual risky behaviors and each characteristic is denoted by: * *p* < 0.05; ** *p* < 0.01; *** *p* < 0.001.

**Table 2:** Ordinary Least Squares Regression Models Predicting Past Year Sexual Risk Behaviors among Homeless Adults (*n* = 460).

Independent Variables	Condomless Sex		New Sexual Partner		Multiple Sexual Partners	
	OR	95% CI	OR	95% CI	OR	95% CI
HIV Risk Perceptions						
Low Perceived HIV risk	0.27	(0.18–0.42) ***	0.29	(0.15–0.55) ***	0.23	(0.11–0.49) ***
High Perceived HIV risk	1.00	-	1.00	-	1.00	-
Age Characteristics						
Age of Sexual Activity Initiation						
≤13 years	1.34	(0.80–2.26)	0.93	(0.58–1.51)	1.10	(0.41–2.92)
≥14 years	1.00	-	1.00	-	1.00	-
Age at First Homeless						
≤24 years	1.45	(0.94–2.25)	1.17	(0.62–2.21)	1.80	(1.09–2.98) *
≥25 years	1.00	-	1.00	-	1.00	-
Current Age						
≤24 years	2.48	(0.97–6.29)	3.65	(2.21–6.02) ***	2.08	(1.09–3.98) *
≥25 years	1.00	-	1.00	-	1.00	-
Covariates						
Sex						
Male	1.00	-	1.00	-	1.00	-
Female	2.10	(1.33–3.34) **	1.18	(0.67–2.08)	0.80	(0.58–1.11)
Marital Status						
Single	1.00	-	1.00	-	1.00	-
Married	3.83	(1.98–7.39) ***	0.45	(0.28–0.73) **	0.76	(0.27–2.13)
Education						
<High School Diploma	1.35	(0.83–2.21)	1.06	(0.72–1.57)	1.01	(0.56–1.82)
High School Diploma or more	1.00	-	1.00	-	1.00	-
Race/Ethnicity						
White	1.00	-	1.00	-	1.00	-
Minority Race/Ethnicity	1.13	(0.77–1.64)	1.04	(0.76–1.43)	1.00	(0.52–1.92)
Sexually Transmitted Infections						
Present	2.07	(1.29–3.31) **	1.63	(0.97–2.73)	2.19	(0.96–5.01)
Absent	1.00	-	1.00	-	1.00	-
Sexual Orientation						
Heterosexual	1.00	-	1.00	-	1.00	-
LGBTQ	0.82	(0.61–1.12)	1.48	(0.82–2.66)	2.23	(1.37–3.62) **
Perceived Stress						
Low Stress	1.00	-	1.00	-	1.00	-
Moderate to Severe	1.07	(0.81–1.43)	0.87	(0.57–1.33)	0.67	(0.40–1.10)
Alcohol or Substance Use Disorder Diagnoses						
No	1.00	-	1.00	-	1.00	-
Yes	0.94	(0.71–1.24)	1.49	(1.03–2.17) *	0.68	(0.50–0.91) **

* *p* < 0.05; ** *p* < 0.01; *** *p* < 0.001.
